# The Mitochondrial Genome of the Sea Anemone *Stichodactyla haddoni* Reveals Catalytic Introns, Insertion-Like Element, and Unexpected Phylogeny

**DOI:** 10.3390/life11050402

**Published:** 2021-04-28

**Authors:** Steinar Daae Johansen, Sylvia I. Chi, Arseny Dubin, Tor Erik Jørgensen

**Affiliations:** 1 Faculty of Biosciences and Aquaculture, Nord University, 8049 Bodø, Norway; aaduu@protonmail.com (A.D.); tor.e.jorgensen@nord.no (T.E.J.); 2 Department of Medical Biology, Faculty of Health Sciences, UiT—The Arctic University of Norway, 9037 Tromsø, Norway; 3 Centre for Innovation, Canadian Blood Services, Ottawa, ON K1G 4J5, Canada; sylvia.ighemchi@blood.ca

**Keywords:** Actiniaria, group I intron, mtDNA, mitogenome, phylogeny, rearrangement, sea anemone

## Abstract

A hallmark of sea anemone mitochondrial genomes (mitogenomes) is the presence of complex catalytic group I introns. Here, we report the complete mitogenome and corresponding transcriptome of the carpet sea anemone *Stichodactyla haddoni* (family Stichodactylidae). The mitogenome is vertebrate-like in size, organization, and gene content. Two mitochondrial genes encoding NADH dehydrogenase subunit 5 (ND5) and cytochrome c oxidase subunit I (COI) are interrupted with complex group I introns, and one of the introns (ND5-717) harbors two conventional mitochondrial genes (ND1 and ND3) within its sequence. All the mitochondrial genes, including the group I introns, are expressed at the RNA level. Nonconventional and optional mitochondrial genes are present in the mitogenome of *S. haddoni*. One of these gene codes for a COI-884 intron homing endonuclease and is organized in-frame with the upstream COI exon. The insertion-like *orfA* is expressed as RNA and translocated in the mitogenome as compared with other sea anemones. Phylogenetic analyses based on complete nucleotide and derived protein sequences indicate that *S. haddoni* is embedded within the family Actiniidae, a finding that challenges current taxonomy.

## 1. Introduction

Hexacoral mitochondria harbor economically organized and vertebrate-like mitochondrial genomes (mitogenomes) ranging in size from 16 to 22 kb [1]. Most mitogenomes consist of a single circular DNA coding for the same set of two ribosomal RNAs (rRNAs) and 13 hydrophobic oxidative phosphorylation (OxPhos) proteins as compared with that of vertebrates [2]. More than 200 complete hexacoral mitogenome sequences are available representing all five extant orders, i.e., Actiniaria (sea anemones), Zoantharia (colonial anemones), Corallimorpharia (mushroom corals), Antipatharia (black corals), and Scleractinia (stony corals) [1]. Sequencing analyses indicate frequent occurrence of nonconventional and optional genes, as well as a highly reduced tRNA gene repertoire [2,3,4,5,6,7]. Insertion-like *orfA* is a representative of a widespread nonconventional mitochondrial gene in sea anemones, but its RNA transcript or derived protein has, so far, not been linked to a cellular function [5,8]. The most unusual feature, however, is the presence of complex group I introns [1,9].

Group I introns are mobile genetic elements found in a variety of genetic compartments, including mitochondria [10,11]. Mitochondrial introns in metazoans are scarce and restricted to only a few orders within the Cnidaria, Porifera, or Placozoa phyla [2,12]. Group I introns catalyze their own splicing reaction at the RNA level by a ribozyme consisting of conserved RNA paired segments (named P1 to P9) organized into catalytic, substrate, and scaffold helical stack domains [13,14,15].

The RNA splicing reaction is well studied [16] and initiated by an exogenous guanosine (exoG) cofactor associated with the P7 paired segment, which subsequently attacks and cleaves RNA at the 5’ splice site (SS) within segment P1. The exoG becomes covalently ligated to the 5’ end of the intron RNA. In the second step of splicing, the terminal intron nucleotide (ωG) replaces exoG in P7 and becomes attacked by the free 3′ hydroxyl group of the upstream exon. The result of splicing is intron excision and exon ligation. Some group I introns carry homing endonuclease genes (HEGs) that promote genetic mobility at the DNA level [15,17,18]. Homing endonucleases are sequence specific DNases that cleave an intron-lacking allele of the host gene, resulting in intron spread by homing. There are several distinct families of HEGs, and mobile group I introns in mitochondria encode homing endonucleases of the LAGLIDADG family [17].

Two mitochondrial genes are found interrupted by complex group I introns in hexacorals. The NADH dehydrogenase subunit 5 gene (ND5) contains an intron at position 717 (human ND5 gene numbering [19]). The ND5-717 intron is obligatory, strictly vertically inherited, and has a fungal origin [19]. ND5-717 varies dramatically in size between hexacoral orders due to a large P8 insertion containing 2–15 conventional OxPhos genes. The shortest form, which is represented by most sea anemone species, corresponds to a P8 insertion of the ND1 and ND3 genes. Both group I intron cis-splicing (most sea anemones) and back-splicing (mushroom corals) appear involved in ND5-717 RNA processing [1,7]. The cytochrome c oxidase subunit I (COI) gene contains mobile-type introns at three different genic positions (720, 867, or 884; human COI gene numbering [19]). Different hexacoral orders tend to harbor COI introns representing distinct evolutionary histories [1,20,21], and sea anemones carry COI-884 group I introns containing LAGLIDADG-type HEGs within P8 [5,8,20].

The family Stichodactylidae consists of only of two genera, *Heteractis* and *Stichodactyla*, and about 10 species [22]. Carpet sea anemones of the genus *Stichodactyla* have a distinct morphology with flattened column and very short tentacles that complicates taxonomy based on external features [23]. Phylogenetic analyses based on partial ribosomal RNA gene sequences from nuclei and mitochondria indicate a relationship between *Stichodactyla* and members of the family Actiniidae [22,24]. Here, we report the complete mitogenome and corresponding transcriptome of the tropical sea anemone *Stichodactyla haddoni*. We characterize two complex catalytic group I introns and provide support of a mitogenome rearrangement involving the nonconventional mitochondrial *orfA*. Mitogenome-based phylogeny indicates that *S. haddoni* is embedded within the family Actiniidae.

## 2. Materials and Methods

### 2.1. Animal Collection and Nucleic Acid Isolation

A specimen of Haddon’s Carpet Anemone (*Stichodactyla haddoni*) of Indo-Pacific origin was retrieved, in 2013, from a pet shop (Tromsø, Norway) and kept alive for several years in our in-house reef tank at the University of Tromsø (UiT) (Tromsø, Norway). Genomic DNA was isolated from approximately 20 mg of fresh tissue containing tentacles, column, and oral disc using the Wizard Genomic DNA Purification kit (Promega, Madison, WI, USA), according to the manufacturer’s instructions. Tissue was homogenized in 600 µL nuclei lysis solution for 20 s at 6000 rpm using Precellys 24 homogenizer (Stretton Scientific, Stretton, UK), and polysaccharide contaminants were removed by phenol/chloroform extraction steps. The purified DNA was eluted in water. Total RNA was isolated using the TRIzol reagent (Thermo Fisher Scientific, Waltham, MA, USA), as previously described [6]. Approximately 30 mg fresh tissue containing tentacles, column, and oral disc was crushed directly in liquid nitrogen, and then in Trizol using the same ”Precellys” settings as described above for DNA isolation. The RNA was purified from each tissue separately by repeated chloroform extractions, followed by precipitation in isopropanol overnight at 4 °C. The RNA pellet was washed in 70% ethanol and resuspended in nuclease-free water (Thermo Fisher Scientific, Waltham, MA, USA).

### 2.2. DNA and RNA Sequencing

Genomic DNA and total RNA were subjected to whole genome and transcriptome Ion Personal Genome Machine (Ion PGM) sequencing, essentially as previously described [6]. In short, all library preparations, template reactions, and sequencing steps were performed according to the standard protocols. Ion Xpress ^TM^ Plus gDNA Fragment Library Preparation kit (Thermo Fisher Scientific, Waltham, MA, USA) was used for DNA library preparation. Approximately 1 µg input genomic DNA was physically sheared for 400 bp selection on a Covaris S2 sonicator (Woburn, MA, USA). The whole transcriptome library was constructed using an Ion Total RNA-Seq Kit v2. Total RNA was polyA-selected using the mRNA DIRECT Purification Kit (Thermo Fisher Scientific, Waltham, MA, USA) and subsequently fragmented enzymatically. The fragmented total RNA was subjected to reverse transcription with a reverse transcriptase mix (Invitrogen 10X SuperScript^®^ III Enzyme Mix, Thermo Fisher Scientific, Waltham, MA, USA). Final preparations and sequencing were performed using the Ion PGM™ Sequencing 400 Kit (Thermo Fisher Scientific, Waltham, MA, USA ), according to the manufacturer’s protocol and on 316 v2 chips. Selected mtDNA regions, including non-coding intergenic regions, were subjected to PCR amplification, plasmid cloning, and Sanger sequencing using specific primers, essentially as previously described [6,8].

### 2.3. Mitogenome Assembly and Annotation

The mitogenome sequence of *S. haddoni* was assembled from the whole genome read pools. The initial sequence was built using MIRA assembler v3.4.1.1 [25] with the *Urticina eques* mitochondrial genome (HG423144) as a reference [5]. Remaining reads were subsequently mapped interatively to the initial sequence using Mitochondrial baiting and iterative mapping (MITObim) script v1.6 [26] with default settings. Mitogenome annotation was performed using MITOS revision 272 [27], supported by manual correction of coding sequences.

### 2.4. Mitochondrial Transcriptome

Transcriptome data were generated for *S. haddoni* and *S. helianthus*. Mitochondrial mRNAs in sea anemones are polyadenylated [5,8]. Transcripts from *S. haddoni* were unambiguously determined, using the corresponding mitogenome sequence (MW760873) as reference, by analysis of quality-filtered Ion PGM reads on CLC Genomics Workbench v8.5 (CLC-Bio, Aarhud, Denmark). About 15.45 million poly(A) enriched RNA reads were obtained, corresponding to 5.90 million reads from oral disk, 5.60 million reads from tentacles, and 3.95 million reads from column; 8530 reads (0.055%) were unambiguously identified as mitochondrial transcripts. Transcriptome data of *S. helianthus*, obtained by Illumina paired reads, was retrieved from the NCBI SRA database (SRR7126073) [28]. About 42.68 million poly(A) enriched RNA reads were obtained, and 12,053 reads (0.028%) were unambiguously identified as mitochondrial transcripts when compared to the *S. haddoni* mitogenome sequence (MW760873). The Illumina paired reads were mapped by “STAR” version 2.7 (https://pubmed.ncbi.nlm.nih.gov/23104886/ (accessed on 1 March 2021)).

### 2.5. Phylogenetic Analysis

The sequences were aligned with T-COFFEE v11.00 using t_coffee_msa, mafft_msa, muscle_msa parameters. Alignments for each gene were created and trimmed independently, and then concatenated to make the final sequence. All internal gaps were included. A general time reversible substitution model with discrete gamma distribution for rate heterogeneity across sites (GTRGAMMA) was applied to all partitions. Phylogenetic analyses were conducted using MEGA X software [29] and RAxML version 8.2.12 for ML trees. All sequence alignments were model tested prior to tree constructions. Maximum-likelihood (ML), neighbor joining (NJ), and minimal evolution (ME) methods were used for comparison. The topologies of the trees were evaluated by 500 bootstrap replicates.

## 3. Results

### 3.1. Characteristic Features of the S. Haddoni Mitogenome

The complete circular mitogenome (mtDNA) sequence of the *S. haddoni* sea anemone (18.999 bp, GenBank accession number MW760873) was determined on both strands using a combined Ion PGM and Sanger sequencing strategy. The 19 annotated mitochondrial genes (Table S1) were all located on the same strand and correspond to two rRNA genes (encoding SSU and LSU rRNAs), two tRNA genes (encoding tRNA^fMet^ and tRNA^Trp^), and 15 protein coding genes. The latter group consisted of 13 conventional mitochondrial genes coding for OxPhos proteins common among most metazoan mitogenomes, and two nonconventional mitochondrial protein genes located within intron and intergenic regions (IGR).

**Figure 1:**
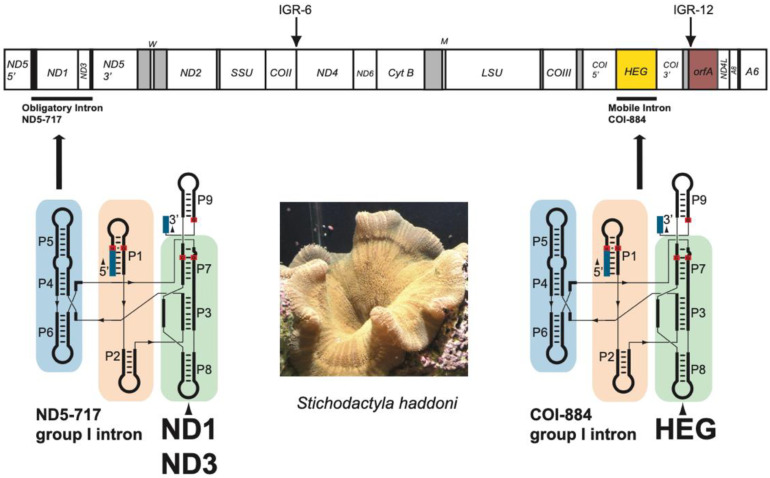
Mitogenome features in *Stichodactyla haddoni*. Gene content and organization of the circular mitogenome presented as a linear map. The mitogenome harbors 15 protein coding genes, two rRNA genes, and 2 tRNA genes. All genes are encoded by the same DNA strand. SSU and LSU, mitochondrial small- and large-subunit rRNA genes. The tRNA genes M and W (tRNA^fMet^ and tRNA^Trp^) are indicated by the standard one-letter symbols for amino acids. ND1-6, NADH dehydrogenase subunit 1–6 genes; COI-III, cytochrome c oxidase subunit I-III genes; Cyt B, cytochrome b gene; A6 and A8, ATPase subunit 6 and 8 genes; HEG, homing endonuclease gene; *orfA*, open reading frame A gene; IGR-6 and -12, intergenic regions 6 and 12. The ND5-717 and CO-884 introns are schematically indicated below the gene map as paired segment (P1 to P9) catalytic RNA core folds. Segment P8 contains large insertions; ND1 and ND3 genes in ND5-717, and a HEG in COI-884. Photo of *Stichodactyla haddoni* specimen by S.D. Johansen.

All conventional mitochondrial genes, including rRNA and tRNA genes, were organized in a similar order as compared with that of most sea anemone mitogenomes investigated [1], and a linear map of the circular *S. haddoni* mtDNA is presented in Figure 1. Some interesting and unusual features were noted. Firstly, the ND5 gene was interrupted by a complex group I intron (ND5-717) harboring the genes of ND1 and ND3 within its structure. Secondly, the COI gene was intervened by a mobile-like group I intron (COI-884) containing a homing endonuclease gene (HEG). Finally, the nonconventional mitochondrial *orfA* found within IGR-6 in other sea anemones (Table S2) was translocated to IGR-12 in *S. haddoni*.

### 3.2. Two Complex Catalytic Group I Introns

**Figure 2:**
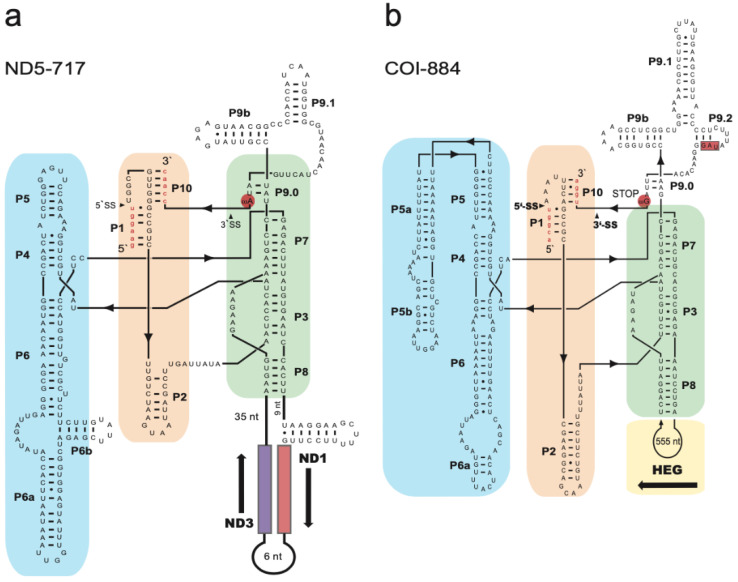
Secondary structure diagram of *Stichodactyla haddoni* mitochondrial group I introns. (**a**) Secondary structure of the ND5-717 group I ribozyme (segments P1 to P10). Flanking ND5 exon sequences shown in red lowercase letters. 5′SS and 3′SS indicate splice sites. The three helical stacks, named Scaffold, Substrate, and Catalytic domains are indicated by blue, yellow, and green boxes, respectively. The last nucleotide of the intron (ω), which is considered to be a universally conserved guanosine (ωG) among group I introns, is ωA in ND5-717 (red circle). The P8 segment harbors the ND1 and ND3 genes. (**b**) Secondary structure of the COI-884 group I ribozyme (segments P1 to P10). Flanking COI exons sequences are in red lowercase letters. The last intron nucleotide (ωG) is indicated (red circle). The three helical stacks are indicated by blue, yellow, and green boxes. The P8 extension contains the HEG insertion. Note that the HEG stop codon (UAG, red box) is located close to the 3′ end of the intron sequence.

**Figure 3:**
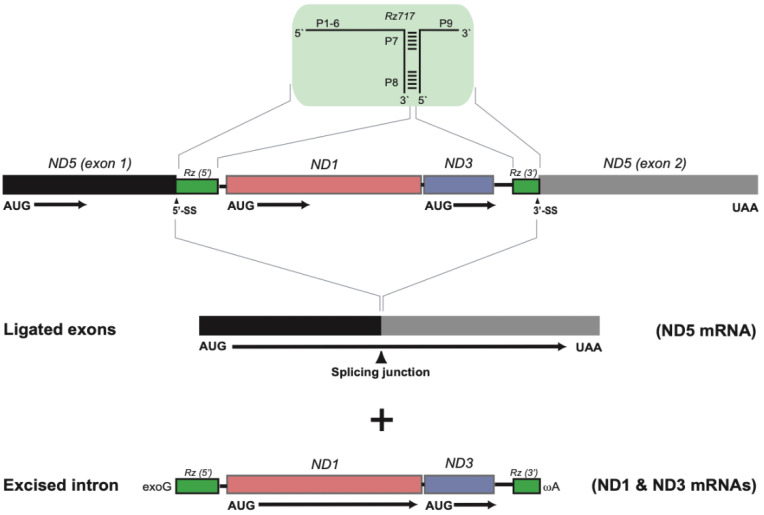
*Stichodactyla haddoni* ND5-717 intron splicing. The group I ribozyme (Rz717, green box) is indicated above the precursor map, and ligated ND5 mRNA is shown below. The ND1 and ND3 mRNAs are proposed generated from the excised intron. Initiation codon (AUG) and stop codon (UAA) of the ND5 mRNA, as well as the splicing junction, are indicated. exoG, exogenous guanosine cofactor; ωA, 3′ terminal intron nucleotide.

Two group I introns were present at conserved sites within the ND5 gene (site 717) and COI gene (site 884). Secondary structure folding of the corresponding RNA confirmed both introns as group I introns (Figure 2). Two interesting structural features were noted in the *S. haddoni* ND5-717 intron (Figure 2a). The terminal nucleotide of the intron, which is catalytical important and universally conserved as a guanosine (ωG) in group I introns, was replaced by ωA. The P8 segment was found to contain a large insertion harboring two OxPhos genes (ND1 and ND3 genes). Splicing of ND5-717 results in a ligated ND5 mRNA and the excised intron RNA is proposed to generate the mRNA precursors of ND1 and ND3 (Figure 3).

**Figure 4:**
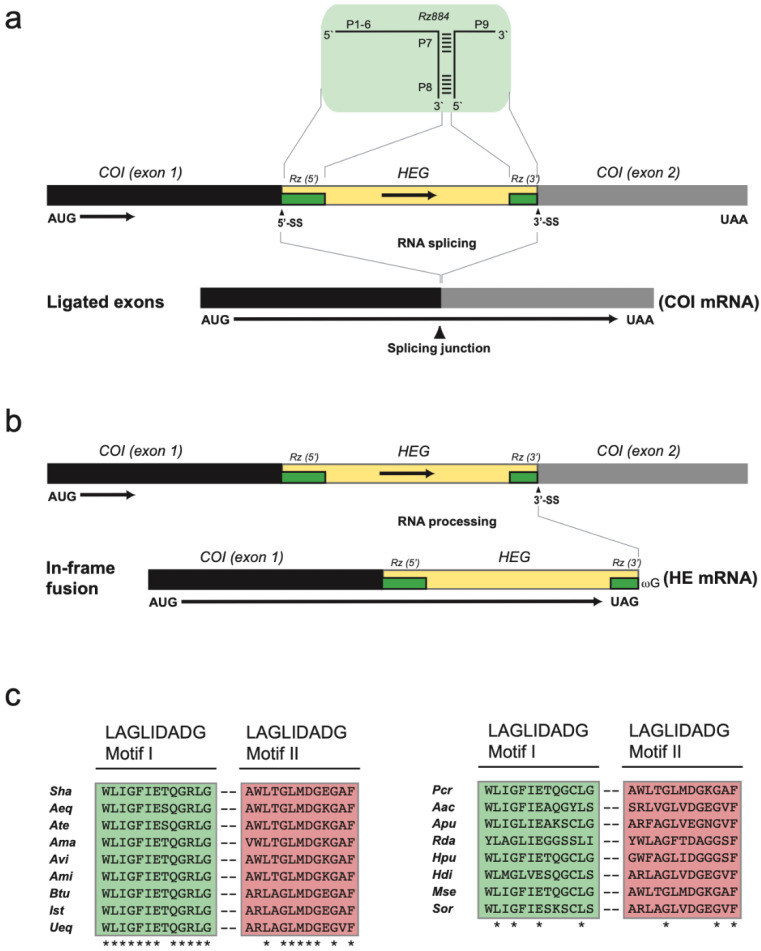
*Stichodactyla haddoni* COI-884 intron splicing and processing. (**a**) The group I ribozyme (Rz884, green box) is indicated above the precursor map, and ligated COI mRNA is shown below. Start (AUG) and stop (UAA) codons are indicated. HEG, homing endonuclease gene. (**b**) Intron RNA processing at the 3′ splice site (SS) generates a COI-HEG in-frame fusion product probably important for HEG expression. HE mRNA, homing endonuclease mRNA. (**c**) LAGLIDADG amino acid sequence motif of the COI-884 encoded homing endonucleases in sea anemones. Each endonuclease contains two copies of the sequence motif (green and red boxes). Left panel, alignment of *Stichodactyla haddoni* (*Sha*) to eight Actinidae species (*Aeq*, *Actinia equina*; *Ate*, *Actinia tenebrosa*; *Ama*, *Anemonia majano*; *Avi*, *Anemonia viridis*; *Ami*, *Anthopleura midori*; *Btu*, *Bolocera tuediae*; *Ist*, *Isosicyonis striata*; *Ueq*, *Urticina eques*). Right panel, alignment of *Phymanthus crucifer* (*Pcr*) to species representing seven different families (*Aac*, *Antholoba achates*; *Apu*, *Aiptasia pulchella*; *Rda*, *Relicanthus daphneae*; *Hpu*, *Halcampoides purpurea*; *Hdi*, *Hormathia digitata*; *Mse*, *Metridium senile*; *Sor*, *Sagartia ornata*). *, identical amino acid residues in alignments. See Table S2 for accession numbers and taxonomy.

The mobile-like COI-884 intron is optional among sea anemones and noted in 81% of inspected species [1]. The secondary structure fold indicates a typical group IC1 intron with a complex back-folded P5 segment (Figure 2b). RNA splicing restores the COI mRNA by exon ligation (Figure 4a). The *S. haddoni* COI-884 intron was found to harbor a homing endonuclease gene (HEG) in segment P8, which expands its 5’ and 3′ ends into the entire intron. Thus, the COI-884 intron sequence has a dual coding potential of a homing endonuclease and a catalytic RNA.

### 3.3. Nonconventional Protein Coding Genes

**Figure 5:**
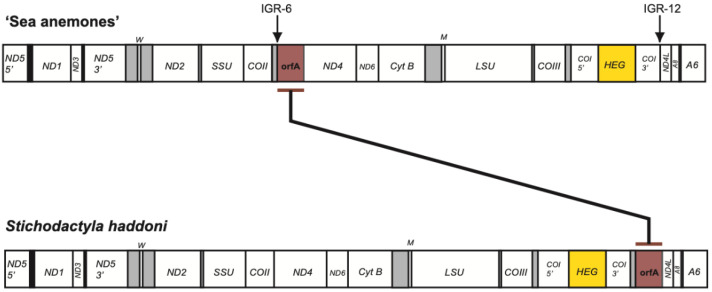
Translocation of mitochondrial *orfA* in the *Stichodactyla haddoni*. The *orfA* is located within IGR-6 in all sea anemones investigated, except IGR-12 in *S. haddoni*. See legend to Figure 1 for mitochondrial gene abbreviations.

Two nonconventional protein coding genes were noted in *S. haddoni*, i.e., the intron HEG and *orfA*. A closer inspection of the COI-884 intron HEG revealed an in-frame fusion to the upstream COI exon, indicating a COI-HEG fusion strategy in gene expression (Figure 4b). Furthermore, the *S. haddoni* homing endonuclease belongs to the LAGLIDADG family (Figure 4c), a homing endonuclease family common among mitochondrial and chloroplast mobile introns [17,18]. The gene *orfA* has been previously noted among several sea anemone mitogenomes and suggested to be an insertion-like element [5,8]. The derived protein sequence appeared conserved among sea anemones inspected, and orfA in *S. haddoni* was clearly similar to the C-terminal part of corresponding orfA in *Anthopleura midori* and *U. eques* (Figure S1). Interestingly, *orfA* in *S. haddoni* is located in IGR-12 (between the COI and ND4L genes), which is different from that in other sea anemone mitogenomes (IGR-6, between genes of COII and ND4) (Figure 5 and Table S2).

### 3.4. Expression of Mitochondrial Genes

**Figure 6:**
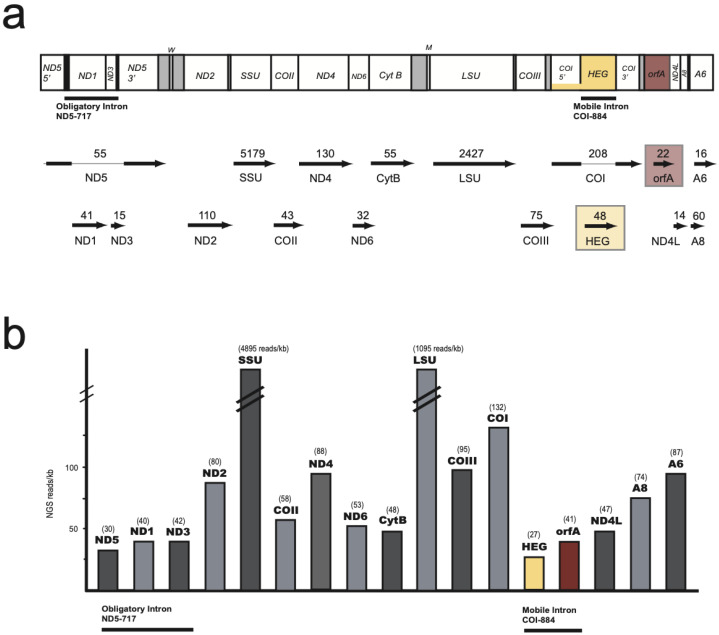
Mitochondrial transcripts from next generation sequencing (NGS) libraries in *Stichodactyla haddoni*. (**a**) Mapping of Ion PGM reads from protein coding and rRNA coding regions. Libraries were based on the poly (A) fraction of total cellular RNA isolated from oral disc, tentacles, and column. Read coverage per gene region is presented below the mitogenome organization map. mRNAs from the nonconventional mitochondrial genes are highlighted in boxes. (**b**) Histograms of estimated normalized read numbers (NGS reads/kb). See legend to Figure 1 for mitochondrial gene abbreviations.

An Ion PGM sequencing approach was used to assess mitochondrial transcripts from *S. haddoni*. The analysis showed that all protein genes were expressed as RNA in all tissue samples assessed (oral disc, tentacles, and column), including the HEG and *orfA,* as well as ligated ND5 and COI mRNAs (Figure 6). The fraction of mitochondrial reads was at 0.055% of total reads, corresponding to 0.074% in oral disc, 0.011% in tentacles, and 0.096 in column. The majority of mitochondrial transcripts, however, was represented by rRNAs (89%). This corresponds well to that observed in other sea anemone species [5,8,30]. To expand the mitochondrial transcriptome assessments, mitochondrial-derived transcripts were mined out from a recently obtained transcriptome dataset in the closely related *S. helianthus* [28]. Illumina paired reads were mapped to the *S. haddoni* mitogenome and normalized against gene sizes (Figure S2a). Similar results were obtained in *S. helianthus* and *S. haddoni*. All conventional and nonconventional mitochondrial genes were clearly expressed as RNA. Both introns were spliced out perfectly, as identified by multiple read sequences representing ligated ND5 mRNA (Figure S2b) and ligated COI mRNA (Figure S2c), and RNA ligation junction consistent with COI-884 full-length circles was observed.

### 3.5. Mitogenome-Based Phylogeny

**Figure 7:**
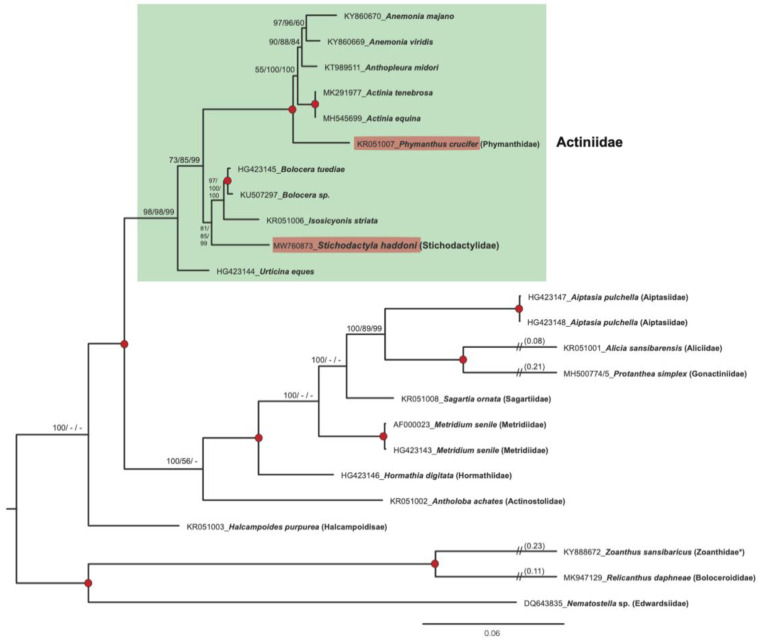
Phylogeny of sea anemones assessed by mitogenome gene sequences. Maximum-likelihood (ML) phylogenetic tree is shown based on alignments of 13,702 nucleotide positions obtained from concatenated genes. Bootstrap values (%) of 500 replications for alternative phylogenetic inference methods are shown at the internal nodes (ML/NJ/ME). Red-filled circles indicate highly significant branch points (bootstrap values of 100%) across the ML, NJ, and ME tree construction methods. Members of the Actiniidae family, including *Stichodactyla haddoni* and *Phymanthus crucifer* are marked by a green box. NJ, neighbor joining; ME, minimal evolution.

To investigate phylogenetic relationships of *S. haddoni* and other sea anemones we performed mitogenome analyses based on concatenated OxPhos genes (13,702 nucleotide positions) and derived protein (3237 amino acid residues) sequences from 23 specimens representing 20 species and 13 Actiniaria families (Table S2). Phylogenetic reconstructions using the ML, NJ, and ME tree-building methods resolved the genera with strong statistical support, and a representative ML tree based on mitochondrial gene sequences is presented in Figure 7. *S. haddoni* (family Stichodactylidae) was found embedded within the family Actiniidae, supported by high bootstrap values by maximum-likelihood (98%), neighbor joining (98%), and minimal evolution (99%) in the nucleotide-based analysis. A similar observation was made for *Phymanthus crucifer* (family Phymanthidae). These unexpected findings were further supported by protein-based phylogenetic reconstructions (Figure S3).

## 4. Discussion

Here, we report the complete mitogenome sequence of the sea anemone *S. haddoni* and corresponding transcriptome and identify several interesting features. These include two complex group I introns, a homing endonuclease gene in fusion with upstream exon, and a translocated *orfA* insertion-like element. Finally, we present a mitogenome-based phylogeny in apparent conflict with current morphology-based taxonomy.

The ND5-717 intron has a sequence organization similar to most other sea anemones [1]. A unique feature, however, is that the last intron nucleotide in ND5-717 is ωA, and not ωG as in most group I introns. A plausible explanation is that ωA prevents 3′ splice site hydrolysis and intron circularization [31], which are RNA processing events that could challenge exon ligation and the generation of ND5 mRNA. An implication of the in-frame COI-HEG fusion strategy is that almost the complete COI-884 intron has protein coding potential, some overlapping with ribozyme coding. A similar strategy has been seen in some introns from fungal mitochondria [32,33]. High-level expression of a homing endonuclease can potentially be hazardous to host genomes due to start activity [17], and downregulation is essential. We observe RNA consistent with COI-884 full-length circles, but a low level of intact fusion transcripts in transcriptome data. This indicates that intron RNA circularization, initiated by 3′ splice site hydrolysis [31], could be involved in downregulation of homing endonuclease expression. Additional experiments on cellular RNAs and appropriate in vitro self-splicing assays are need in order to evaluate this possibility. 

The *orfA* sequence represents a widespread nonconventional sea anemone mitochondrial gene, but with unknown cellular function (Table S2). There are several features that link *orfA* to insertion-like elements, such as being located in highly transcribed regions, has a common but sporadic distribution among species, and its expression is induced by environmental stress [8]. Furthermore, *orfA* appears to be evolving under relaxed selection as compared with most OxPhos genes [5]. Here, we report *orfA* in both *S. haddoni* and *S. helianthus* to be expressed as polyadenylated RNA at similar levels to that of most Complex I (NADH dehydrogenase subunit) genes and associated with a gene translocation event from IGR-6 (typical in sea anemones) to IGR-12. Mitochondrial gene rearrangements have been well documented in hexacoral orders [1]. Deep-water species of stony corals (*Madrepora* and *Lophelia*) [19,34] and mushroom corals (*Corallimorphus* and *Corynactis*) [35] show gene order rearrangements as compared with other members of the orders. A dramatic rearrangement is seen in the *Protanthea* deep-water sea anemone [30]. The mitogenome consists of two distinct mitochromosomes and the gene order is heavily scrambled as compared with that of other investigated sea anemones. Interestingly, both strands contain mitochondrial genes, which differ from all other hexacoral mitogenomes.

The sea anemones (Order Actiniaria) represent a large and morphological diverse group of animals divided into approximately 1200 species in 46 families [22]. Several reports have included molecular markers in order to improve the phylogenetic assessments, studies that have applied nuclear and mitochondrial rRNA gene sequences, mitochondrial protein gene sequences, and complete mitogenomes [5,24,30,36,37,38]. The mitogenome-based phylogeny presented in this study supported a close association between *S. haddoni* (family Stichodactylidae) and members of the family Actiniidae (Figure 7 and Figure S3). Actiniidae constitutes the largest family known among the sea anemones with more than 200 species and 44 genera [22], and our findings corroborate earlier reports that the current Actiniidae taxonomy apparently is polyphyletic [22,24]. A similar finding was noted for *P. crucifer* (family Phymanthidae) [24,39,40], which also appears embedded within the family Actiniidae (Figure 7 and Figure S3). An additional molecular observation in the mitogenomes that link *S. haddoni* and *P. crucifer* to members of Actiniidae is the in-fusion expression mode of intron HEG. This feature is almost exclusively observed among sea anemones of the family Actiniidae (Table S2). Furthermore, a comparison of the conservation of LAGLIDADG sequence motifs of COI-884 intron homing endonucleases also suggests a close relationship of *S. haddoni* and *P. crucifer* to members of the family Actiniidae (Figure 4). Thus, there is an obvious need for revisions in sea anemone taxonomy, especially among Actiniidae and closely related species. One possibility is to add more related taxa (e.g., additional species and genus of the Stichodactylidae) to the mitogenome-based analysis. However, phylogenetic assessments based on rRNA or mitogenome sequence molecular markers are apparently not sufficient to resolve high resolution relationships, and we suggest that a genomic-based phylogeny would probably be more appropriate.

## 5. Conclusions

The mitogenome of *S. haddoni* harbors two complex catalytic group I introns, i.e., ND5-717 which carries the conventional ND1 and ND3 mitochondrial genes, and COI-884 which contains a nonconventional HEG. The latter was organized in-frame with the upstream COI exon 1, indicating a gene fusion strategy of intron homing endonuclease expression. A second nonconventional mitochondrial gene in *S. haddoni*, i.e., *orfA*, was found translocated and associated with mitogenome rearrangement. Phylogenetic analysis indicates *S. haddoni* to be embedded within the Actiniidae family, suggesting a polyphyletic origin of Actiniidae, and calls for a revision in sea anemone taxonomy.

## Data Availability

Sequencing data is available in GenBank under the accession number MW760873.

## Supplementary Materials

The following are available online at https://www.mdpi.com/article/10.3390/life11050402/s1, Figure S1: Amino acid alignment of orfA protein in Stichodactyla haddoni (Sha; 174 aa), Anthopleura midori (Ami, 617 aa) and Urticina eques (Ueq, 644 aa). See Table S2 for accession numbers. Identical residues are indicated (*) above aligned sequences, Figure S2: Mitochondrial transcripts from Illumina paired read libraries in Stichodactyla helianthus [28] mapped to the S. haddoni mitogenome sequence (MW760873). (a) Histograms of estimated normalized read numbers (NGS reads/kb). See legends to Figures 1 and 6 for mitochondrial gene abbreviations. (b) Sashimi plot of ND5 splicing junction, consistent with perfect splicing of the ND5-717 intron. 21 reads mapped unambiguously to the ligated sequence region. (c) Sashimi plot of COI splicing junction, consistent with perfect splicing of the COI-884 intron. 100 reads map unambiguously to the ligated sequence region, Figure S3: Phylogeny of sea anemones assessed by mitogenome protein sequences. Maximum-likelihood (ML) phylogenetic tree is shown based on alignments of 3237 amino acid residues derived from concatenated genes. Bootstrap values (%) of 500 replications for alternative phylogenetic inference methods are shown at the internal nodes (ML/NJ/ME). Red-filled circles indicate highly significant branch points (bootstrap values of 100%) across the ML, NJ, and ME tree construction methods. Members of the Actiniidae family, including S. haddoni and Phymanthus crucifer are marked by a green box. NJ, neighbor joining; ME, minimal evolution, Table S1: Annotation of the complete mitogenome sequence of S. haddoni, Table S2: Key features of sea anemone mitogenomes included in this study.

## References

[1] Johansen S.D., Emblem Å., Soto L.A. (2020). Mitochondrial group I introns in hexacorals are regulatory elements. Advances in the Studies of the Bentic Zone.

[2] Osigus H.J., Eitel M., Bernt M., Donath A., Schierwater B. (2013). Mitogenomics at the base of Metazoa. Mol. Phylogenet. Evol..

[3] Flot J.F., Tillier S. (2007). The mitochondrial genome of *Pocillopora* (Cnidaria: Scleractinia) contains two variable regions: The putative D-loop and a novel ORF of unknown function. Gene.

[4] Beagley C.T., Wolstenholme D.R. (2013). Characterization and localization of mitochondrial DNA-encoded tRNA and nuclear DNA-encoded tRNAs in the sea anemone *Metridium senile*. Curr. Genet..

[5] Emblem Å., Okkenhaug S., Weiss E.S., Denver D.R., Karlsen B.O., Moum T., Johansen S.D. (2014). Sea anemones possess dynamic mitogenome structures. Mol. Phylogenet. Evol..

[6] Chi S.I., Johansen S.D. (2017). Zoantharian mitochondrial genomes contain unique complex group I introns and highly conserved intergenic regions. Gene.

[7] Chi S.I., Dahl M., Emblem Å., Johansen S.D. (2019). Giant group I intron in a mitochondrial genome is removed by RNA back-splicing. BMC Mol. Biol..

[8] Chi S.I., Urbarova I., Johansen S.D. (2018). Expression of homing endonuclease gene and insertion-like element in sea anemone mitochondrial genomes: Lesson learned from *Anemonia viridis*. Gene.

[9] Beagley C.T., Okada N.A., Wolstenholme D.R. (1996). Two mitochondrial group I introns in a metazoan, the sea anemone *Metridium senile*: One intron contains genes for subunits 1 and 3 of NADH dehydrogenase. Proc. Natl. Acad. Sci. USA.

[10] Haugen P., Simon D.M., Bhattacharya D. (2005). The natural history of group I introns. Trends Genet..

[11] Nielsen H., Johansen S.D. (2009). Group I introns: Moving in new directions. RNA Biol..

[12] Schuster A., Lopez J.V., Becking L.E., Kelly M., Pomponi S.A., Worheide G., Erpenbeck D., Cardenas P. (2017). Evolution of group I introns in Porifera: New evidence for intron mobility and implications for DNA barcoding. BMC Evol. Biol..

[13] Cech T.R., Damberger S.H., Gutell R.R. (1994). Representation of the secondary and tertiary structure of group I introns. Nat. Struct. Biol..

[14] Vicens Q., Cech T.R. (2006). Atomic level architecture of group I introns revealed. Trends Biochem. Sci..

[15] Hedberg A., Johansen S.D. (2013). Nuclear group I introns in self-splicing and beyond. Mob. DNA.

[16] Cech T.R. (1990). Self-splicing of group I introns. Annu. Rev. Biochem..

[17] Stoddard B.L. (2005). Homing endonuclease structure and function. Q. Rev. Biophys..

[18] Hafez M., Hausner G. (2012). Homing endonucleases: DNA scissors on a mission. Genomics.

[19] Emblem Å., Karlsen B.O., Evertsen J., Johansen S.D. (2011). Mitogenome rearrangement in the cold-water scleractinian coral *Lophelia pertusa* (Cnidaria, Anthozoa) involves a long-term evolving group I intron. Mol. Phylogenet. Evol..

[20] Goddard M.R., Leigh J., Roger A.J., Pemberton A.J. (2006). Invasion and persistence of a selfish gene in the Cnidaria. PLoS ONE.

[21] Celis J.S., Edgell D.R., Stelbrink B., Wibberg D., Hauffe T., Blom J., Kalinowski J., Wilke T. (2017). Evolutionary and biogeographical implications of degraded LAGLIDADG endonuclease functionality and group I intron occurrence in stony corals (Scleractinia) and mushroom corals (Corallimorpharia). PLoS ONE.

[22] Daly M., Brugler M.R., Cartwright P., Collins A.G., Dawson M.N., Fautin D.G., France S.C., McFadden C.S., Opresko D.M., Rodriguez E. (2007). The phylum Cnidaria: A review of phylogenetic patterns and diversity 300 years after Linnnaeus. Zootaxa.

[23] Attaran-Fariman G., Javid P., Shakouri A. (2015). Morphology and phylogeny of the sea anemone *Stichodactyla haddoni* (Cnidaria: Anthozoa: Actiniaria) from Chabahar Bay, Iran. Turk. J. Zool..

[24] Daly M., Chaudhuri A., Gusmao L., Rodriguez E. (2008). Phylogenetic relationships among sea anemones (Cnidaria: Anthozoa: Actiniaria). Mol. Phylogenet. Evol..

[25] Chevreux B., Wetter T., Suhai S. (1999). Genome sequence assembly using trace signals and additional sequence information. German Conf. Bioinform..

[26] Hahn C., Bachmann L., Chevreux B. (2013). Reconstructing mitochondrial genomes directly from genomic next-generation sequencing reads—A baiting and iterative mapping approach. Nucleic Acids Res..

[27] Bernt M., Donath A., Jühling F., Externbrink F., Florentz C., Fritzsch G., Stadler P.F. (2013). MITOS: Improved de novo metazoan mitochondrial genome annotation. Mol. Phylogenet. Evol..

[28] Rivera-de-Torre E., Martinez-del-Pozo A., Garb J.E. (2018). *Stichodactyla helianthus*’ *de novo* transcriptome assembly: Discovery of a new actinoporin isoform. Toxicon.

[29] Kumar S., Stecher G., Tamura K. (2016). MEGA7: Molecular evolutionary genetics analysis version 7.0 for bigger datasets. Mol. Biol. Evol..

[30] Dubin A., Chi S.I., Emblem Å., Moum T., Johansen S.D. (2019). Deep-water sea anemone with a two-chromosome mitochondrial genome. Gene.

[31] Nielsen H., Fiskaa T., Birgisdottir A.B., Haugen P., Einvik C., Johansen S.D. (2003). The ability to form full-length intron RNA circles is a general property of nuclear group I introns. RNA.

[32] Lambowitz A.M., Belfort M. (1993). Introns as mobile genetic elements. Annu. Rev. Biochem..

[33] Guo W.W., Moran J.V., Hoffman P.W., Henke R.M., Butow R.A., Perlman P.S. (1995). The mobile group I intron 3a of the yeast mitochondrial COCI gene codes a 35-kDa processed protein that is an endonuclease but not a maturase. J. Biol. Chem..

[34] Lin M.F., Kitahara M.V., Tachikawa H., Fukami H., Miller D.J., Chen C.A. (2012). Novel organization of the mitochondrial genome in the deep-sea coral, *Madrepora oculate* (Hexacorallia, Scleractinia, Oculinidae) and its taxonomic implications. Mol. Phylogenet. Evol..

[35] Lin M.F., Kitahara M.V., Luo H., Tracey D., Geller J., Fukami H., Miller D.J., Chen C.A. (2014). Mitochondrial genome rearrangements in the Scleractinia/ corallimorpharian complex: Implications for coral phylogeny. Genome Biol. Evol..

[36] Daly M., Gusmao L.C., Reft A.J., Rodriguez E. (2010). Phylogenetic signal in mitochondrial and nuclear markers in sea anemones (Cnidaria, Actiniaria). Integr. Comp. Biol..

[37] Rodriguez E., Barbeitos M.S., Brugler M.R., Crowley L.M., Grajales A., Gusmao L., Haussermann V., Reft A., Daly M. (2014). Hidden among sea anemones: The first comprehensive phylogenetic reconstruction of the order Actiniaria (Cnidaria, Anthozoa, Hexacorallia) reveals a novel group of hexacorals. PLoS ONE.

[38] Titus B.M., Benedict C., Laroche R., Gusmao L.C., Van Deusen V., Chiodo T., Meyer C.P., Berumen M.L., Bartholomew A., Yanagi K. (2019). Phylogenetic relationships among the clownfish-hosting sea anemones. Mol. Phylogenet. Evol..

[39] Zhang L., Zhu Q. (2017). Complete mitochondrial genome of the sea anemone, *Anthopleura midori* (Actiniaria: Actiniidae). Mitochondrial DNA.

[40] Foox J., Brugler M., Siddall M.E., Rodriguez E. (2016). Multiplexed pyrosequencing of nine sea anemone (Cnidaria: Anthozoa: Hexacorallia: Actiniaria) mitochondrial genomes. Mitochondrial DNA.

