# Supplementary material for: The Mitochondrial Genome of the Sea Anemone Stichodactyla haddoni Reveals Catalytic Introns, Insertion-Like Element, and Unexpected Phylogeny
Source: Life (Basel). 2021 Apr 28;11(5):402. doi: 10.3390/life11050402 (PMC8146996; doi:10.3390/life11050402)
Supplement: Supplementary file 1 [file life-11-00402-s001.zip › life-1185781-supplementary.pdf]

Supplementary Materials

The Mitochondrial Genome of the Sea Anemone *Stichodactyla haddoni* Reveals Catalytic Introns, Insertion-like Element, and Unexpected Phylogeny

Steinar D. Johansen [1,2,*], Sylvia I. Chi [3], Arseny Dubin [1], and Tor E. Jørgensen [1]

[1] Faculty of Biosciences and Aquaculture, Nord University, Bodø 8049, Norway; steinar.d.johansen@nord.no (SDJ), aaduu@protonmail.com (AD), tor.e.jorgensen@nord.no (TEJ)

[2] Department of Medical Biology, Faculty of Health Sciences, UiT - The Arctic University of Norway, Tromsø 9037, Norway

[3] Centre for Innovation, Canadian Blood Services, Ottawa, ON K1G 4J5, Canada; sylvia.ighemchi@blood.ca (SIC)

Figure S1: Amino acid alignment of orfA protein in *Stichodactyla haddoni* (Sha; 174 aa), *Anthopleura midori* (Ami; 617 aa) and *Urticina eques* (Ueq; 644 aa). See Table S2 for accession numbers. Identical residues are indicated (*) above aligned sequences.

Figure S2: Mitochondrial transcripts from Illumina paired read libraries in *Stichodactyla helianthus* [Rivera-de-Torre et al. 2018] mapped to the *S. haddoni* mitogenome sequence (MW760873). (a) Histograms of estimated normalized read numbers (NGS reads/ kb). See legends to Figures 1 and 6 in the main manuscript for mitochondrial gene abbreviations. (b) Sashimi plot of ND5 splicing junction, consistent with perfect splicing of the ND5-717 intron. 21 reads mapped unambiguously to the ligated sequence region. (c) Sashimi plot of COI splicing junction, consistent with perfect splicing of the COI-884 intron. 100 reads map unambiguously to the ligated sequence region.

Ref: Rivera-de-Torre, E.; Martinez-del-Pozo, A.; Garb, J.E. *Stichodactyla helianthus*' *de novo* transcriptome assembly: discovery of a new actinoporin isoform. *Toxicon* **2018**, *150*, 105-114. doi:10-1016/j.toxicon.2018.05.014.

Figure S3: Phylogeny of sea anemones assessed by mitogenome protein sequences. Maximum-likelihood (ML) phylogenetic tree is shown based on alignments of 3,237 amino acid residues derived from concatenated genes. Bootstrap values (%) of 500 replications for alternative phylogenetic inference methods are shown at the internal nodes (ML/NJ/ME). Red filled circles indicate highly significant branch points (bootstrap values of 100%) across the ML, NJ and ME tree construction methods. Members of the Actiniidae family, including *Stichodactyla haddoni* and *Phymanthus crucifer* are marked by a green box. NJ, Neighbor Joining; ME, Minimal evolution.

Table S1: Annotation of the complete mitogenome sequence of *S. haddoni*.

Table S2: Key features of sea anemone mitogenomes included in this study.

Figure S1. Amino acid alignment of orfA protein in *S. haddoni* (Sha), *A. midori* (Ami) and *U. eques* (Ueq).

```
        *  * * ***  ***  *****  ***  ***************** ********** **
Ami     MSQLALKNLIKRKWLSVIIISYLFFIVVKSGGDNFICVKVTLLSLAVLLTHYPWLSFSLL
Ueq     MRKLSLRNLIRKKWLGIIIISYFVFIVAISGGDNFICVKVTLLSLAILLTHYPWLSFGLL

        ************************************* *********  **  ****
Ami     IISVLMNLLKGYYLLTIFILFFLIMFRVALYKKVKGIRWLILIYWSALILMWTGTIVISY
Ueq     IISVLMNLLKGYYLLTIFILFFLIMFRVALYKKVKGIWWLILIYWSAFTLMCAGTIVTFC

        ** *    *   * **** **   * * **  * *** ** ****  *************
Ami     WGETLFKFLSYNQIGPEPFGLSMSFAQSEASSFKLAPEVWARPSWIYSVGALSIVEGFAQ
Ueq     WGGTFIEYLPFSQVGPEPLGLHGPFEQNEAPPFSLAPKVWERPSWVSSVGALSIVEGFAQ

        **** ***   *** ***** ************** ** **********  *** ** **
Ami     TPFENMTYLLEVKGLTTGFPSRASLAQVEIDPLISKLESTGLFPFKPSVIENFRGLLKDW
Ueq     TPFEDMTYHLDVKGSTTGFPCRASLAQVEIDPLISRLERTGLFPFKPSVIDNFRDLLMDW

        ******* ****  ** * ** ***  ******  * ***********************
Ami     HCEPLLFLSNFFEAHLSKIALVEEKLQSIGLYQKKLFGLHLYNDWLVLTTYDECTRIGIN
Ueq     HCEPLLFFSNFFDVHLPKLALMEEKCKSIGLYQDTLLGLHLYNDWLVLTTYDECTRIGIN

        **** ***  ******* * ********************* * ******* **** *
Ami     SMTPMGPLDTSQLTDIKIFGSNIKLMFGERQIEIALSNLRETKALGLQELWDNSPPEPFA
Ueq     SMTPTGPLYASQLTDIKAFESNIKLMFGERQIEIALSNLRESKVLGLQELWNNSPP&PNF

           * ***  *    ***   * *** *   **** * ***** *  * *   *   ***
Ami     FDNPNFVHGDLNLHKGPRPNSWLIERFTPPRGSNPFVDSFSQFMGQKVVNVIFPLINNGE
Ueq     YYDPVFVHSELDSFRGPRQDTWAIERYTTSPGSNPLVHSFSQFFGKQVQNILIPPMENGE

        * *     *** ********** *****    ** *  **      **** **** *
Ami     GVLHITLADGSLLSFPVEVKVGNTPSSWSPRLELMPERINFVSPRFFMFLTPPTGFFMPT
Ueq     GILDVHFTDGSFLSFPVEVKVGQTPSSWDFKVELWPDTINIVWADHFMFLKPPTGIFMPN
Sha                                                    MFLTPPTGWFECK

             *** * *** ** *** * ***  *  ** ** ** * **  *  **   *
Ami     NQNILDIPLNWVWGPPLFYKGVITGNPFDFTDDYDHPGALNLTKKITESNIFHDMSVYQT
Ueq     NGTVFDIPLNCVWGAPLYYKGAIAGNPLNFAADYNHPGALNLIKKVTESNIFHDMSLCQT
Sha     EGTFLDIPINFVWGPPLFYKGVIVGDPFNFVSDYKHPAALGLVKKMTETKIFQEMSLFQR

        ** ***  *** *  ** *** * **       * ** *  ***    ***  *  ***
Ami     KGQGQGSQGILKNFETFLKNFNKEELAQCDQLPPVIKLPREELFSRGYIQPSAAVRLWLY
Ueq     KGQGQGDIGILRNFKTFWKNFNKKELARCNEAHPMIKIPRQELFRMGHIQPMTALKLWLY
Sha     KGEGQGDIGILKNYKTFLKNFIKKELSRSSTVPPFIKIPWKELFLGEYIQPITALKLWLW

                                      * **  *
Ami     ----------------------------SLRQQILKSEGSCQVSP
Ueq     RGKLLSPNTSYELARKALSLPKKLFIVQSFRRQILKSGPSSDES
Sha     RGELYSSDTSYGYACKVLGLPKNQFIIRAFRRQIVRSNQIS
```

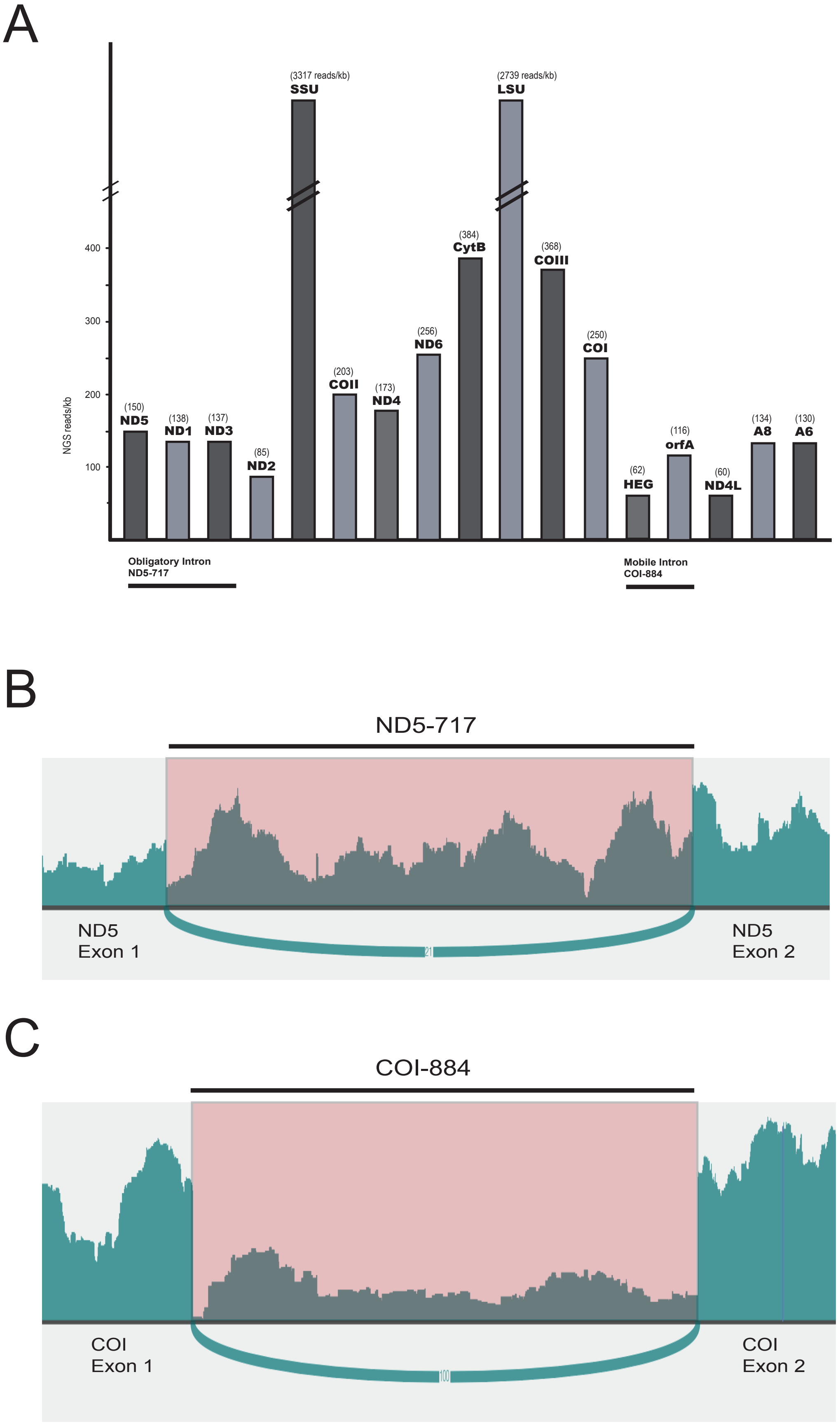
A
NGS reads/kb
100
200
300
400
(3317 reads/kb)
SSU
(2739 reads/kb)
LSU
(150)
ND5
(138)
ND1
(137)
ND3
(85)
ND2
(203)
COII
(173)
ND4
(256)
ND6
(384)
CytB
(368)
COIII
(250)
COI
(62)
HEG
(116)
orfA
(60)
ND4L
(134)
A8
(130)
A6
Obligatory Intron
ND5-717
Mobile Intron
COI-884
B
ND5-717
ND5
Exon 1
ND5
Exon 2
C
COI-884
COI
Exon 1
COI
Exon 2

Figure S2

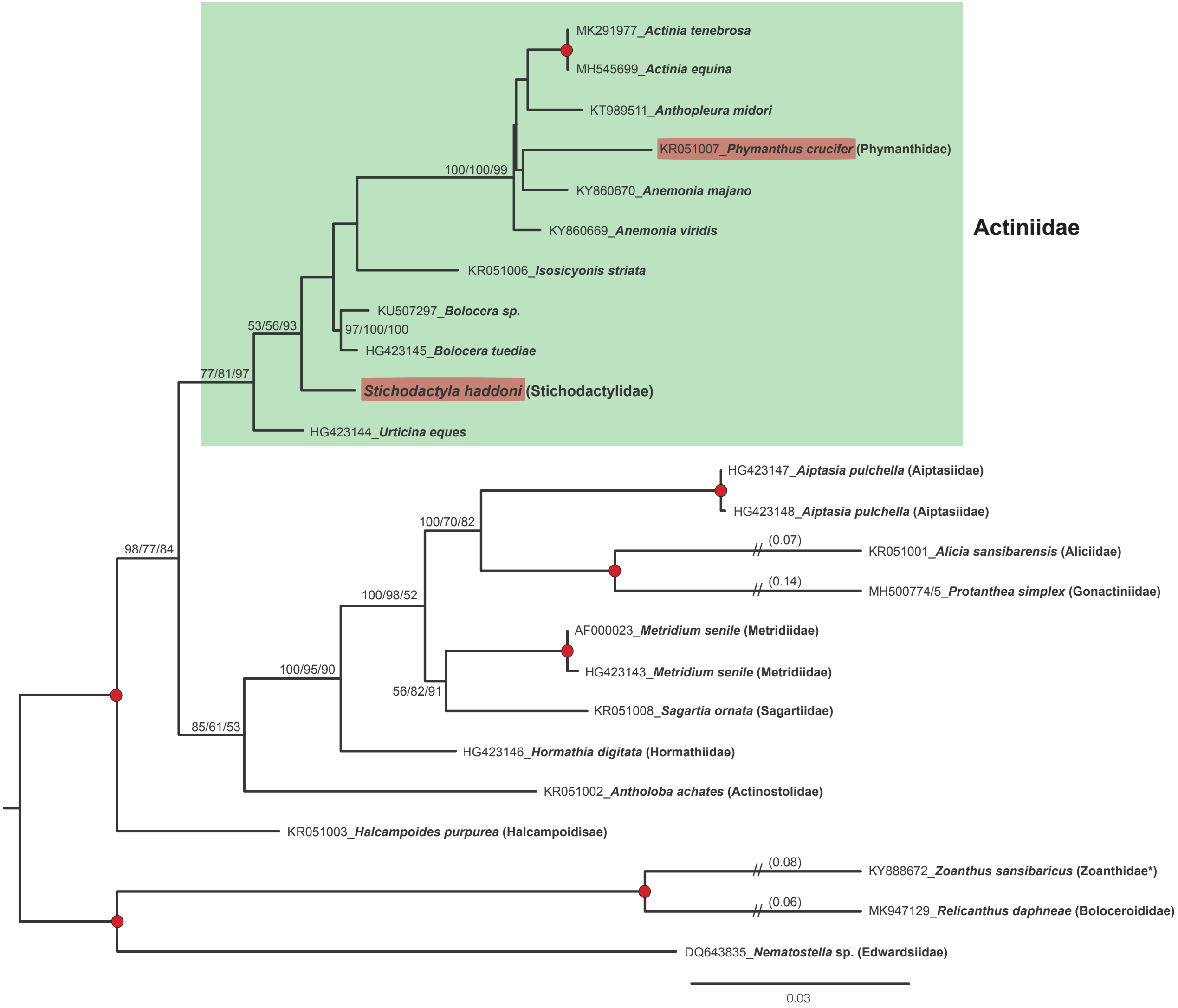
MK291977_Actinia tenebrosa
MH545699_Actinia equina
KT989511_Anthopleura midori
KR051007_Phymanthus crucifer (Phymanthidae)
100/100/99
KY860670_Anemonia majano
KY860669_Anemonia viridis
Actiniidae
KR051006_Isosicyonis striata
KU507297_Bolocera sp.
53/56/93
97/100/100
HG423145_Bolocera tuediae
77/81/97
Stichodactyla haddoni (Stichodactylidae)
HG423144_Urticina eques
HG423147_Aiptasia pulchella (Aiptasiidae)
HG423148_Aiptasia pulchella (Aiptasiidae)
100/70/82
(0.07)
KR051001_Alicia sansibarensis (Aliciidae)
98/77/84
(0.14)
MH500774/5_Protanthea simplex (Gonactiniidae)
100/98/52
AF000023_Metridium senile (Metridiidae)
100/95/90
HG423143_Metridium senile (Metridiidae)
56/82/91
KR051008_Sagartia ornata (Sagartiidae)
85/61/53
HG423146_Hormathia digitata (Hormathiidae)
KR051002_Antholoba achates (Actinostolidae)
KR051003_Halcampoides purpurea (Halcampoidisae)
(0.08)
KY888672_Zoanthus sansibaricus (Zoanthidae*)
(0.06)
MK947129_Relicanthus daphneae (Boloceroididae)
DQ643835_Nematostella sp. (Edwardsiidae)
0.03

Figure S3

Table S1: Annotation of *Stichodactyla haddoni* complete mitochondrial genome

Genes & Regions	Pos	Size nt	Size aa
ND5-exon 1	1-717	717	239
ND5-717, 5' ribozyme	718-940	223	
ND1	941-1924	984	327
IGR-1	1925-1930	6	
ND3	1931-2287	357	118
ND5-717, 3' ribozyme	2288-2397	111	
ND5-exon 2	2398-3513	1116	371
ND5		1833	610
IGR-2	3514-3809	296	
W	3810-3879	70	
IGR-3	3380-3993	614	
ND2	3994-5376	1383	461
IGR-4	5377-5425	49	
SSU	5426-6483	1058	
IGR-5	6484-6535	52	
COII	6536-7282	741	246
IGR-6	7283-7287	5	
ND4	7288-8763	1467	488
IGR-7	8764-8767	4	
ND6	8768-9376	609	202
IGR-8	9377-9403	27	
CytB	9404-10555	1152	383
IGR-9	10556-11033	418	
M	11034-11104	71	
LSU	11105-13321	2248	
IGR-10	13322-13392	71	
COIII	13393-14181	789	262
IGR-11	14182-14349	176	
COI-exon 1	14350-15242	893	
Intron COI-884	15243-16107	865	
HEG[1]	14350-16095	1746	581
COI-exon 2	16108-16789	682	
COI		1575	524
IGR-12	16790-17718	285	
OrfA	17196-17729	534	177
ND4L	17719-18018	300	99
IGR-13	18019-18043	24	
A8	18043-18258	216	71
IGR-14	18259-18290	33	
A6	18291-18980	690	229
IGR-15	18981-18999	19	

[1] Reading frame fused to COI 5' exon

Table S2. Key features of sea anemone mitogenomes included in this study

A: Sea anemones (Order Actiniaria)

Species	Acc.	Mt Size	COI intron	HEG fusion	orfA
Family Actiniidae					
Actinia equina	MH545699	20,690 bp	COI+	-	+ (IGR-6)
Actinia tenebrosa	MK291977	20,691 bp	COI+	-	+ (IGR-6)
Anemonia majano	KY860670	19,545 bp	COI+	+ (pseudo)	+ (IGR-6)
Anemonia viridis	KY860669	20,108 bp	COI+	+ (pseudo)	+ (IGR-6)
Anthopleura midori	KT989511	20,039 bp	COI+	+ (pseudo)	+ (IGR-6)
Bolocera tuediae	HG423145	19,143 bp	COI+	+ (in frame)	-
Bolocera sp.	KU507297	19,463 bp	COI+	+ (in frame)	-
Isosicyonis striata	KR051006	19,001 bp	COI+	+ (in frame)	+ (IGR-6)
Urticina eques	HG423144	20,458 bp	COI+	+ (in frame)	+ (IGR-6)
Family Actinostolidae					
Antholoba achates	KR051002	17,816 bp	COI+	+ (in frame)	+ (IGR-6)
Family Aiptasiidae					
Aiptasia pulchella [4]	HG423147	19,791 bp	COI+	-	+ (IGR-6)
Aiptasia pulchella [4]	HG423148	19,790 bp	COI+	-	+ (IGR-6)
Family Aliciidae					
Alicia sansibarensis	KR051001	19,575 bp	COI-	-	-
Family Boloceroididae					
Relicanthus daphneae	MK947129	17,727 bp	COI+	+ (in frame)	-
Family Edwardsiidae					
Nematostella sp.	DQ643835	16,389 bp	COI-	-	-
Family Gonactiniidae					
Protanthea simplex	MH500774/75	21,326 bp	COI-	-	-
Family Halcampoidisae					
Halcampoides purpurea	KR051003	18,038 bp	COI+	-	-
Family Hormathiidae					
Hormathia digitata	HG423146	18,754 bp	COI+	-	+ (IGR-6)
Family Metridiidae					
Metridium senile	HG423143	17,444 bp	COI+	-	-
Metridium senile	AF000023	17,743 bp	COI+	-	-
Family Phymanthidae					
Phymanthus crucifer	KR051007	19,727 bp	COI+	-	+ (IGR-6)
Family Sagartiidae					
Sagartia ornata	KR051008	17,446 bp	COI+	-	-
Family Stichodactylidae					
Stichodactyla haddoni	Unpublished [5]	18,999 bp	COI+	+ (in frame)	+ (IGR-12)

B: Colonial anemones (Order Zoantharia)

Species	Acc. no	Mt Size	COI intron	HEG fusion	orfA
Family Zoanthidae					
Zoanthus sansibaricus	KY888672	20,972 bp	COI+	-	-

Notes: 1) COI+ or -, COI intron present or absent. 4) The sea anemone *Aiptasia pulcella* may also be annotated as *Exaiptasia pallida.* 5 Information from our unpublished complete mitochondtial genome sequence of *Stichodactyla haddoni.*
